# Draft Genome Sequence of Three Antibiotic-Resistant *Leuconostoc mesenteroides* Strains of Dairy Origin

**DOI:** 10.1128/genomeA.01018-15

**Published:** 2015-09-10

**Authors:** Ilenia Campedelli, Ana Belén Flórez, Elisa Salvetti, Susana Delgado, Luigi Orrù, Luigi Cattivelli, Ángel Alegría, Giovanna E. Felis, Sandra Torriani, Baltasar Mayo

**Affiliations:** a Department of Biotechnology, University of Verona, Italy; b Departamento de Microbiología y Bioquímica, Instituto de Productos Lácteos de Asturias (IPLA-CSIC), Spain; c Consiglio per la ricerca in agricoltura e l’analisi dell’economia agraria, Genomics Research Centre, Fiorenzuola d'Arda, Piacenza, Italy

## Abstract

*Leuconostoc mesenteroides* is a lactic acid bacterium (LAB) commonly associated with fermented foods. Here, we report the genome sequence of three selected dairy strains, showing atypical antibiotic resistances (AR). Genome analysis provided a better understanding of the genetic bases of AR in *Leuconostoc* and its potential transferability among foodborne bacteria.

## GENOME ANNOUNCEMENT

*Leuconostoc mesenteroides* is a lactic acid bacterium (LAB) species commonly found in association with food substrates, both of plant and animal origin (1–3). In the dairy industry, strains of this species are naturally present as contaminants in many traditional cheese varieties or they are deliberately added as adjunct cultures (4–6). Indeed, their capacity to produce aromatic compounds, such as acetaldehyde, acetoin, and diacetyl, in addition to lactic and acetic acid, carbon dioxide, and dextrans, contribute to the development of desirable sensory traits of dairy products (7–10). An increasing number of *L. mesenteroides* genome sequences have been deposited in the GenBank database (11–13), including those of *L. mesenteroides* subsp. *cremoris* ATCC 19254^T^ (ACKV00000000) and of two strains isolated from dairy starter cultures (6, 14).

Here, we report the genome sequence of three *L. mesenteroides* strains isolated from Italian soft cheese samples, namely, *L. mesenteroides* subsp. *dextranicum* LbE15, *L. mesenteroides* subsp. *mesenteroides* LbE16, and *L. mesenteroides* subsp. *cremoris* LbT16. These strains displayed atypical resistance to erythromycin and clindamycin (LbE15), kanamycin, streptomycin, tetracycline, and virginiamycin (LbE16), and tetracycline (LbT16).

Whole-genome sequencing was performed using the Illumina HiSeq2000 platform at the Beijing Institute of Genomics (BIG) (Beijing, China) with a paired-end library. Quality of reads was verified using the FastQC software and *de novo* assembly was performed with the SPAdes Assembler version 3.5.0 (15). The genome sequences of the three *Leuconostoc* strains were annotated by the National Center for Biotechnology Information (NCBI) Prokaryotic Genome Annotation Pipeline. Genome information for each strain is reported in Table 1.

**TABLE 1.** Whole-genome information of the three *L. mesenteroides* strains LbE15, LbE16, and LbT16

Strain	Genome size (bp)	*N*_50_ (bp)	G+C content (%)	No. of genes	No. of coding sequences	No. of pseudo genes	Accession no.
LbE15	2,008,120	76,771	37	2,044	1,900	90	LAYN00000000
LbE16	2,036,196	160,323	37	2,100	1,949	97	LAYU00000000
LbT16	1,906,463	753,66	37	1,939	1,713	172	LAYV00000000

Totals of 1,524,191, 1,682,147, and 1,416,327 paired-end reads (2- × 75-bp length on average) were assembled into 65, 86, and 66 contigs for strain LbE15, LbE16, and LbT16, respectively (genome coverage of about 200×). The lengths of the largest assembled contigs were 259,998 bp, 285,382 bp, and 382,195 bp for the genome of LbE15, LbE16, and LbT16, respectively. The three genomes contain 53 genes encoding RNAs, of which 3 are for rRNAs and 50 for tRNAs.

Preliminary analysis of the sequences revealed the presence of *erm*(B) in LbE15 and *tet*(S) in LbE16, coding for erythromycin (16) and tetracycline (17) resistance, respectively. However, these genotypes only partially explain the resistance phenotypes, and further studies will be necessary to gain a complete overview of the genetic background required for AR.

Increasing attention is paid to the presence of AR genes and their possible transferability to other species and eventually to pathogens (18). Therefore, the complete genomes of the three *L. mesenteroides* strains reported here represent a fundamental starting point to improve the current knowledge regarding the molecular basis of AR in LAB and to evaluate its transference capability via horizontal gene transfer among food-borne bacteria.

### Nucleotide sequence accession numbers. 

The whole-genome shotgun projects of the *L. mesenteroides* strains have been deposited in DDBJ/EMBL/GenBank under the accession numbers reported in Table 1. The versions described in this paper are the first versions, LAYN01000000 (*L. mesenteroides* subsp. *dextranicum* LbE15), LAYU01000000 (*L. mesenteroides* subsp. *mesenteroides* LbE16), and LAYV01000000 (*L. mesenteroides* subsp. *cremoris* LbT16).
